# Uncommon Occurrence of Compound Nevus in the Oral Cavity

**DOI:** 10.1155/crid/2505039

**Published:** 2026-05-22

**Authors:** Akash Nhemaphuki, Manoj Humagain, Arjun Hari Rijal, Simant Lamichhane, Sachita Thapa, Sujan Timsina

**Affiliations:** ^1^ Department of Periodontology and Oral Implantology, Kathmandu University School of Medical Sciences, Nepal, kusms.edu.np

**Keywords:** biopsy, case report, gingiva, nevus

## Abstract

**Introduction:**

Compound nevi are less common. Compound nevi account for approximately 5.9%–16.5% of all reported oral melanocytic nevi. Current evidence suggests no clear association with patient age or sex. The oral compound nevus is most typically found on the hard palate and buccal mucosa, with fewer cases occurring elsewhere in the mouth. This case report aims to highlight the rare case of a compound nevus in the gingiva of the maxillary anterior region of a patient who presented for cosmetic enhancement.

**Case Presentation:**

A 55‐year‐old healthy female patient reported with a chief complaint of discoloration of gum in the upper front region of the jaw. On intraoral examination, there was a bluish‐black‐brown color of gingiva with respect to the right maxillary central and lateral incisors. The area was asymptomatic and non‐scrapable and had a slightly irregular texture on palpation. The patient had no similar pigmented lesions elsewhere, denied smoking or alcohol use, and reported no family history of nevi in the same region. An orthopantomogram was taken. An excisional biopsy was carried out under local anesthesia. Histological examination revealed a compound nevus. Follow‐up after 5 months shows 2 mm gingival recession with respect to the right central incisor.

**Conclusion:**

Postmenopausal women with a compound nevus—although these lesions are typically small and asymptomatic, histopathological evaluation is essential to ensure accurate diagnosis and patient safety.

## 1. Introduction

In the study of Buchner, Merrell, and Carpenter, the oral melanocytic nevi accounted for only 0.1% of 89,430 oral biopsies analyzed over 19 years [1]. About 5.9%–16.5% of all reported oral melanocytic nevi are compound nevus [2]. As less common [3], this lesion represents benign melanocytic proliferations that originate from basal‐layer melanocytes, typically developing early in life [4]. As these lesions mature, melanocytes aggregate at the epithelial–connective tissue junction and gradually extend into the underlying stroma, without exhibiting vascular or lymphatic invasion. This pattern of maturation produces a characteristic dome‐shaped lesion [4]. Most reported cases of oral compound nevi occur on the hard palate (33.3%–57.1%) (Table 1), making it the predominant location in published series [4]. In comparison, the gingiva where the present lesion was found accounts for only 8.3%–14.3% of cases, highlighting its relative rarity [4]. Oral melanocytic nevi are most often identified incidentally during routine examinations, as they rarely produce symptoms [7]. Biopsied compound nevi are more frequently reported in female patients, possibly reflecting heightened cosmetic awareness [8].

**Table 1:** Prevalence of compound nevi in various part of oral cavity in different populations.

Study	Location (%)				
	Hard palate	Buccal mucosa	Gingiva	Labial mucosa	Retromolar pad
Buchner and Hansen [5], 1987 (12 cases)	33.3%	41.7%	8.3%	8.3%	8.3%
Buchner et al. [6], 1990 (130 cases)	41.7%	33.3%	8.3%	8.3%	8.3%
Buchner et al. [1], 2004 (15 cases)	33.3%	33.3%	13.3%	20%	—
Meleti et al. [7], 2007 (7 cases)	57.1%	28.6%	14.3%	—	—

In a study in Nepal, there were two cases (9.4%) of nevi, one intradermal nevus and one compound nevus [9]. This distribution further underscores the unusual nature of the present case. Oral melanocytic nevi show a preference for the upper oral cavity; a region also associated with the highest occurrence of oral melanoma [6]. This overlap has raised the possibility that a subset of oral melanocytic nevi may undergo malignant transformation. More than 40% of oral melanomas arise on the hard or soft palate, while the maxillary gingiva where the present lesion was located accounts for approximately 16% of cases [10]. This case report aims to highlight the rare case of a gingival compound nevus in the maxillary anterior region of a patient who presented for cosmetic enhancement.

## 2. Case Report

**Figure 1:**
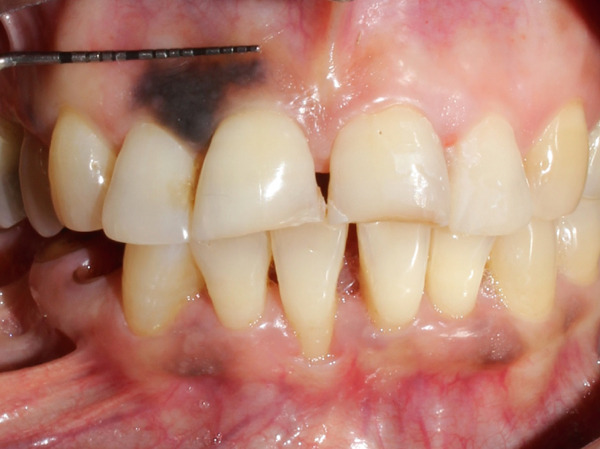
Initial clinical presentation with University of North Carolina 15 mm periodontal probe measuring 10 mm mesio‐distal diameter of lesion.

**Figure 2:**
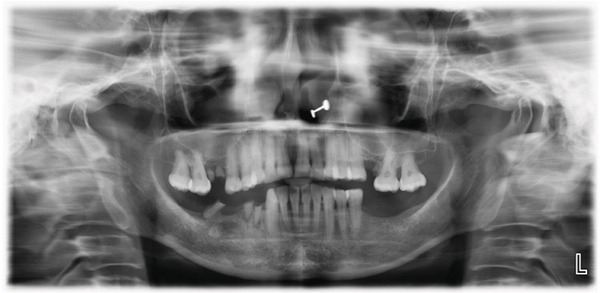
Radiographic presentation shown in Orthopantomogram.

**Figure 3:**
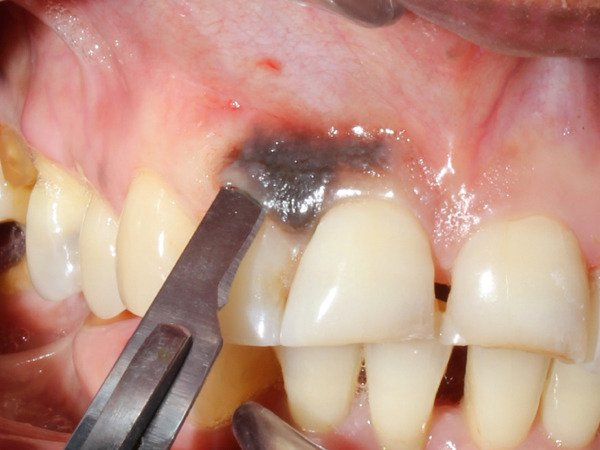
Crevicular incision was made around the involved teeth with number 15 blade with Bard–Parker handle.

**Figure 4:**
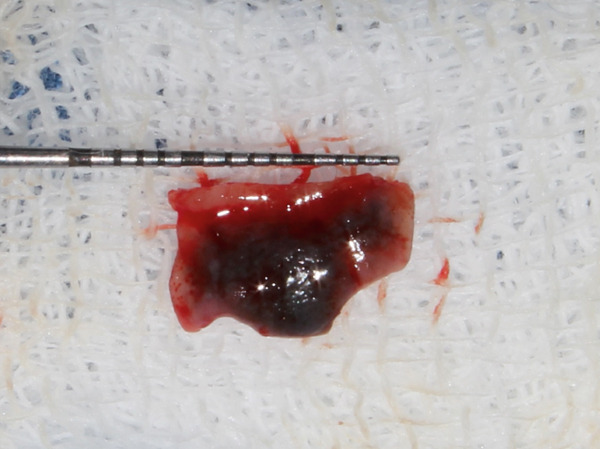
Excised tissue length with University of North Carolina 15 mm periodontal probe placed on sterilized moistened gauge.

**Figure 5:**
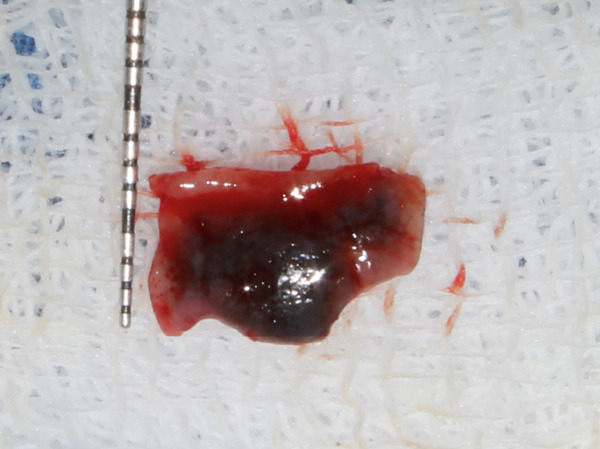
Excised tissue breadth with University of North Carolina 15 mm periodontal probe placed on sterilized moistened gauge.

**Figure 6:**
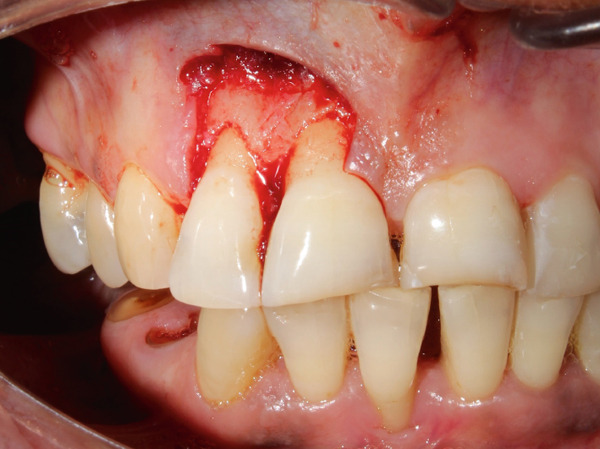
Wound after excision exposing coronal third of root and alveolar bone with respect to right maxillary central and lateral incisors.

**Figure 7:**
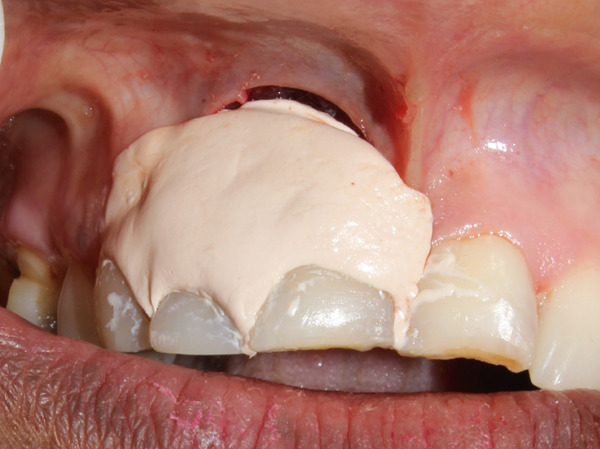
Periodontal dressing of wound.

A 55‐year‐old post‐menopausal woman from Butwal, Nepal reported to the Department of Periodontology and Oral Implantology at Kathmandu University School of Medical Sciences, Dhulikhel Hospital, Dhulikhel, Kavrepalanchok, Nepal with a chief complaint of discolored gum in right upper front region of jaw for a year and wished to have it removed for a better smile. She visited Lumbini Provincial Hospital, a government hospital in Butwal, Lumbini Province, for a check‐up 6 months ago, but she did not receive any treatment at that time. A review of her medical history revealed no significant systemic illnesses. She looked healthy while her visit with no abnormal posture and gait. Her blood pressure was in normal range. On intraoral examination, the lesion appeared bluish black to brown located with respect to right maxillary central and lateral incisors, measuring roughly 10 mm mesiodistally measured using University of North Carolina 15 mm periodontal probe, and extended from the marginal gingiva to the mucogingival junction (Figure 1). It showed mild elevation and merged smoothly with the adjacent gingiva. The area was asymptomatic, non‐scrapable, and had a slightly irregular texture on palpation. The patient had no similar pigmented lesions elsewhere, denied smoking or alcohol use, and reported no family history of nevi in the same region. An orthopantomogram was taken for diagnostic purposes to complete the radiographic evaluation of the jawbone and dentition (Figure 2). A comprehensive treatment plan was thoroughly discussed with the patient, and written informed consent was obtained prior to procedure. Before undergoing a procedure, the patient was asked to gargle with 10 mL of 0.2% chlorohexidine mouthwash (CHX Oral Rinse 100 mL Asian Pharmaceuticals Pvt. Ltd., HF4F+95Q, Siddhartha Hwy, Padsari 32900) for 30 s. A single use 26‐gauge hypodermic needle (Hi‐Tech Medics Pvt. Ltd.) with 3‐mL syringe (Lifeline Disposable Syringe—3mL 23G 1″) was used. Local anesthesia was achieved using an infiltration technique in relation to the maxillary central and lateral incisors with 2% lidocaine containing epinephrine (Lidokam Injection, 2% w/v, 30 mL, Kamla Life Science Ltd.), ensuring adequate hemostasis and patient comfort. A planned full‐thickness (mucoperiosteal) flap was executed. Initially, a crevicular incision was made around the involved teeth (Figure 3), extending to adjacent teeth to provide adequate surgical access. The flap was then carefully elevated using a periosteal elevator, reflecting both the mucosa and periosteum to expose the underlying alveolar bone. Following flap reflection, an excisional biopsy of the lesion was performed using a No. 15 blade (Lister Surgical Blade Sterile R, India. MFG/MD/2023/00845) mounted on a Bard–Parker handle. Excisional mass was placed on sterilized moistened gauge and measurement was taken (Figures 4, 5). Immediately excisional mass was transferred to 10% neutral buffered formalin for fixation. The surgical site was thoroughly irrigated using sterile normal saline. A wound exposing the alveolar bone was observed (Figure 6), and a periodontal dressing (COE‐PAK Periodontal Dressing, Regular Set, GC America Inc. ALSIP, IL 60803, United States) (Figure 7) was applied to protect the surgical site. Patient was instructed to avoid pulling or inspecting the surgical site. Patient was advised to take pain medication as prescribed (Tab. ibuprofen 400 mg, 1 tab p/o every 6–8 h SOS) before anesthesia (numbness) wears off. Patient was also instructed to avoid brushing and flossing around the surgical area and perform normal hygiene in other areas. Patient was instructed to gargle with 10 mL of 0.2% chlorohexidine mouthwash (CHX Oral Rinse 100 mL, Asian Pharmaceuticals Pvt. Ltd., HF4F+95Q, Siddhartha Hwy, Padsari 32900) for 30 s two times a day for 14 days. Patient was recalled for follow‐up after 10 days to remove periodontal dressing.

**Figure 8:**
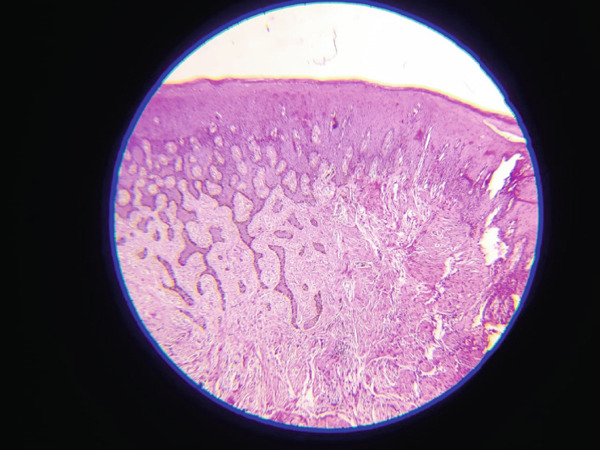
Melanin‐containing nevus cells along the basal epithelial layer and in focal nests within the underlying connective tissue.

**Figure 9:**
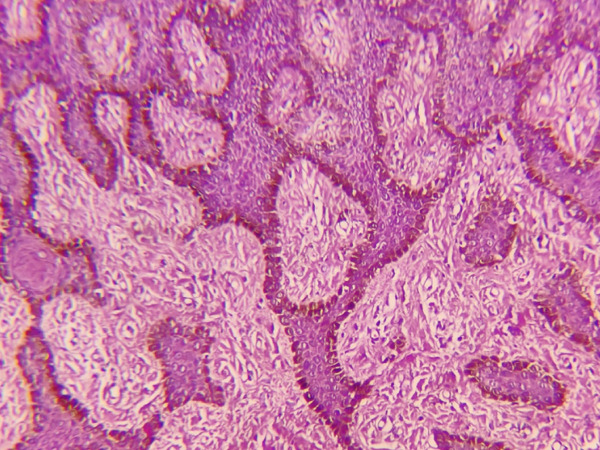
Melanin‐containing nevus cells in focal nests within the underlying connective tissue.

Histological analysis was done in the Department of Oral Pathology in Dhulikhel Hospital by an oral pathologist. Report demonstrated ortho‐keratinized stratified squamous epithelium overlying a fibrous connective tissue matrix. Melanin‐containing nevus cells were observed along the basal epithelial layer and in focal nests within the underlying connective tissue (Figures 8 and 9). These features supported a diagnosis of a compound nevus.

**Figure 10:**
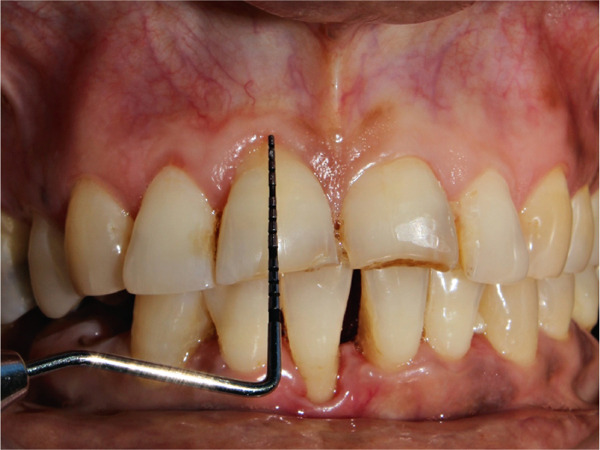
Follow‐up after 5 months shows 2 mm of gingival recession with respect to right maxillary central incisor.

Patient came for follow‐up after 10 days with no any fresh complaint. COE‐PAK dressing was removed. Healing was uneventful with no pain, swelling, and reappearance of melanin pigment. Healing gingival site was slightly red. Oral hygiene instructions were given. Follow‐up after 5 months shows coral pink color of gingiva without any pigmentation. Two millimeters of gingival recession with respect to right central incisor is seen (Figure 10). Patient was satisfied with her treatment as there was no reoccurrence of pigmentation in gingiva and slight recession was irrelevant to her smile.

## 3. Discussion

The earliest documentation of an oral nevus was provided by Ackermann and Field in 1943 [11]. Oral nevi may be congenital or acquired [2]. They are generally classified into four histopathological types: junctional, intramucosal (intralamina propria), compound, and blue nevi, as consistently reported in international literature [12]. Physiologic and pharmacologic alteration of patient’s estrogen and progesterone levels may lead to increased binding of estrogen and progesterone by their nevus cells [13]. A recurring pattern has been observed in some cases, coinciding with pregnancy and lactation, suggesting a possible hormonal influence [14]. Although oral melanocytic nevi may develop at any age, they are most frequently observed in the second and third decades of life [4]. The present case involves a 55 years old post‐menopausal woman, which is outside the commonly reported age range. No racial differences have been found with oral nevi [15]. Oral melanocytic nevi are typically asymptomatic lesions that are detected in conjunction with another primary complaint. Clinically, compound nevi typically appear as small, slightly elevated papules or plaques, though well‐circumscribed macules can also occur. In a large case series by Buchner A and Hansen LS, oral melanocytic nevi were reported to range from 0.1 to 3.0 cm in size [5], with the majority measuring less than 1 cm and commonly occurring on the palate and buccal mucosa [16]. Their pigmentation varies from light to dark brown, and in some cases, lesions may be non‐pigmented. The surface is typically smooth, although a minority may exhibit a rough, papillomatous, or verrucous texture [6, 16]. Consistent with these findings, the present case demonstrated an asymptomatic, sharply demarcated macular lesion measuring 10 × 7 mm, with a smooth surface and dark bluish‐brown pigmentation, although the location in the present case (gingiva) is less commonly reported.

Pigments that may be present in the oral mucosa include melanin, hemosiderin, metallic deposits, and intraoral tattoos. Additionally, blood and circulatory changes can influence the color of oral tissues and should therefore be considered in the differential diagnosis [17]. The differential diagnosis includes oral melanotic macule, physiologic (racial) pigmentation, and amalgam tattoo [2]. Currently, no definitive data exist regarding the malignant potential of oral melanocytic nevi. Although approximately one‐third of oral melanomas are preceded by pigmented lesions of long duration, the specific histological nature of these precursor lesions remains poorly characterized [7]. Darkening of a pigmented nevus is a clinical sign that may indicate a transition from a benign lesion to a malignant one [17]. As all lesions in these reports were excised before malignant transformation could be evaluated, the question of whether any truly represent precursor nevi remains unresolved. Nevertheless, given their rarity, their clinical similarity to early oral melanoma, and their typically small size, complete excision of oral melanocytic nevi is widely recommended [1].

Complications may arise following treatment with simple excision, although they are generally minor. In the present case, a 2‐mm gingival recession was observed at the 5‐month follow‐up. Recurrence of oral melanocytic nevi is exceedingly rare, with only isolated cases reported in the literature [15].

## 4. Limitations

The present case report has certain limitations. A longer follow‐up period would have been valuable to assess long‐term recurrence or potential malignant transformation; however, this was not feasible as the patient was from a different province and was lost to follow‐up. Additionally, the possible influence of hormonal factors, particularly in the postmenopausal state, could not be evaluated. Future studies incorporating hormonal correlation and larger sample sizes are recommended to better understand the pathogenesis and behavior of oral melanocytic nevi.

## 5. Conclusion

A case of 55‐year‐old post‐menopausal woman with an oral compound nevus, one of the rarest pathologies of the oral cavity, located at maxillary anterior has been presented. Excision and histologic examination of all pigmented lesions are recommended. Although these lesions are typically small and asymptomatic, histopathological evaluation is essential to ensure accurate diagnosis and patient safety. Follow‐up is recommended to rule out recurrence of the lesion.

## Funding

No funding was received for this manuscript.

## Ethics Statement

All procedures performed in the study involving human participants were in accordance with the ethical standards of the institutional and/or national research committee and with the 1964 Helsinki Declaration and its later amendments or comparable ethical standards.

## Consent

Written informed consent was obtained from the patient for performing excision of lesion and also for publication of clinical details and accompanying images. The patient understood that efforts would be made to conceal identity, but anonymity cannot be guaranteed.

## Conflicts of Interest

The authors declare no conflicts of interest.

## Data Availability

The data that support the findings of this study are available from the corresponding author upon reasonable request.

## References

[1] Buchner A. , Merrell P. W. , and Carpenter W. M. , Relative Frequency of Solitary Melanocytic Lesions of the Oral Mucosa, Journal of Oral Pathology & Medicine. (2004) 33, no. 9, 550–557, 10.1111/j.1600-0714.2004.00238.x, 2-s2.0-4844230023, 15357676. 15357676

[2] Cardoso L. B. , Consalaro A. , da Silva Santos P. S. , da Silva Sampieri M. B. , and Tinoco-Araujo J. E. , Oral Compound Nevus, Dermatology Online Journal. (2014) 20, no. 2, 1–6, 10.5070/D3202021542. 24612575

[3] Aguirre A. , Alawi F. , and Tapia J. L. , Glick M. , Pigmented Lesions of the Oral Mucosa, Burket′s Oral Medicine, 2015, 12th edition, People’s Medical Publishing House, 123–146.

[4] Eversole L. R. , Greenberg M. S. and Glick M. , Pigmented Lesions of the Oral Mucosa, Burket’s Oral Medicine—Diagnosis and Treatment, 2003, 10th edition, Hamilton: BC Decker, 10.1093/ortho/30.4.346.

[5] Buchner A. and Hansen L. S. , Pigmented Nevi of the Oral Mucosa: A Clinicopathologic Study of 36 New Cases and Review of 155 Cases From the Literature: Part I: A Clinicopathologic Study of 36 New Cases, Oral Surgery, Oral Medicine, Oral Pathology. (1987) 63, no. 5, 566–572, 10.1016/0030-4220(87)90229-5, 2-s2.0-0023237292, 3473378. 3473378

[6] Buchner A. , Leider A. S. , Merrell P. W. , and Carpenter W. M. , Melanocytic Nevi of the Oral Mucosa: A Clinicopathologic Study of 130 Cases From Northern California, Journal of Oral Pathology & Medicine. (1990) 19, no. 5, 197–201, 10.1111/j.1600-0714.1990.tb00825.x, 2-s2.0-0025084747, 2359037. 2359037

[7] Meleti M. , Mooi W. J. , Casparie M. K. , and Van Der Waal I. , Melanocytic Nevi of the Oral Mucosa—No Evidence of Increased Risk for Oral Malignant Melanoma: An Analysis of 119 Cases, Oral Oncology. (2007) 43, no. 10, 976–981, 10.1016/j.oraloncology.2006.11.013, 2-s2.0-34948901860, 17258496. 17258496

[8] Maize J. C. and Foster G. , Age-Related Changes in Melanocytic Naevi, Clinical and Experimental Dermatology. (1979) 4, no. 1, 49–58, 10.1111/j.1365-2230.1979.tb01590.x, 2-s2.0-0018417638, 445877. 445877

[9] Pudasaini S. and Baral R. , Oral Cavity Lesions: A Study of 21 Cases, Journal of Pathology of Nepal. (2011) 1, no. 1, 49–51, 10.3126/jpn.v1i1.4452.

[10] Hicks M. J. and Flaitz C. M. , Oral Mucosal Melanoma: Epidemiology and Pathobiology, Oral Oncology. (2000) 36, no. 2, 152–169, 10.1016/S1368-8375(99)00085-8, 2-s2.0-0034163123, 10745167. 10745167

[11] Weathers D. R. and Waldron C. A. , Intraoral Cellular Nevi, Oral Surgery, Oral Medicine, Oral Pathology. (1965) 20, no. 4, 467–475, 10.1016/0030-4220(65)90239-2, 2-s2.0-50549219021, 5212792. 5212792

[12] Comerford T. E. , de la Pava S. , and Pickren J. W. , Nevus of the Oral Cavity, Nevus of the oral cavityOral Surgery, Oral Medicine, Oral Pathology. (1964) 17, no. 2, 148–151, 10.1016/0030-4220(64)90132-X, 2-s2.0-43049085764. 14117351

[13] Ellis D. L. and Wheeland R. G. , Increased Nevus Estrogen and Progesterone Ligand Binding Related to Oral Contraceptives or Pregnancy, Journal of the American Academy of Dermatology. (1986) 14, no. 1, 25–31, 10.1016/S0190-9622(86)70002-9, 2-s2.0-0022647162. 3950111

[14] Dyer P. V. and Eveson J. W. , Recurrent Compound Naevus of Gingiva, Journal of Periodontology. (1993) 64, no. 8, 739–741, 10.1902/jop.1993.64.8.739, 2-s2.0-0027650935, 8410613. 8410613

[15] Patil K. and Mahesh K. P. , Oral Myiasis - A Case Report With Clinical, Radiographic and Entomological Findings from Puducherry Union Territory, Southern India, Journal of Indian Academy of Oral Medicine and Radiology. (2009) 21, no. 4, 187–191, 10.4103/0972-1363171069.

[16] Buchner A. and Hansen L. S. , Pigmented Nevi of the Oral Mucosa: A Clinicopathologic Study of 36 New Cases and Review of 155 Cases From the Literature: Part II: Analysis of 191 Cases, Oral Surgery, Oral Medicine, Oral Pathology. (1987) 63, no. 6, 676–682, 10.1016/0030-4220(87)90370-7, 2-s2.0-0023160352, 3473394. 3473394

[17] Akamine R. N. , Compound Nevus of the Buccal mucosa, Oral Surgery, Oral Medicine, and Oral Pathology. (1962) 15, no. 1, 27–33, 10.1016/0030-4220(62)90059-2, 2-s2.0-50549190138, 13859891. 13859891

